# Bilateral Iris Atrophy after the Femtosecond Assisted Laser In Situ Keratomileusis Surgery

**DOI:** 10.1155/2015/127806

**Published:** 2015-06-14

**Authors:** Kenan Olcay, Akin Cakir, Sercan Koray Sagdic, Eyup Duzgun, Yildiray Yildirim

**Affiliations:** ^1^Department of Ophthalmology, Gumussuyu Military Hospital, 34100 Besiktas, Istanbul, Turkey; ^2^Department of Ophthalmology, Golcuk Military Hospital, 41650 Golcuk, Kocaeli, Turkey; ^3^Department of Ophthalmology, Gulhane Military Medical Academy Haydarpasa Training Hospital, 34668 Uskudar, Istanbul, Turkey

## Abstract

*Purpose*. To report an unknown complication of laser in situ keratomileusis (LASIK) surgery. *Case Presentation*. A 28-year-old female presented with photophobia and glare to our eye service. She stated in her medical history that she had undergone femtosecond assisted LASIK surgery in both eyes 15 months ago and her symptoms started just after this surgery. On admission, her best-corrected visual acuity was 10/10 in both eyes. She had mydriatic pupils with no direct light reflex. Examination of the anterior segment revealed bilateral iris atrophy projecting within the LASIK ablation zone and a transillumination defect was remarkable on the slit lamp examination. *Conclusion*. We hypothesized that this condition may have been caused by the abnormally increased IOP that resulted in ischemia in the iris vascular plexus during the suction process of surgery.

## 1. Introduction

Refractive surgery has undergone significant progress and evolution during the past two decades with the advent of the excimer laser. Excimer laser refractive surgical options mainly include photorefractive keratectomy (PRK) and laser in situ keratomileusis (LASIK) [1]. Since FDA approval in 2000, the femtosecond laser has revolutionized the creation of flaps for LASIK and is being used confidently at the present time. Herein we report an interesting and previously unreported complication of this procedure.

## 2. Case Presentation

**Figure 1:**
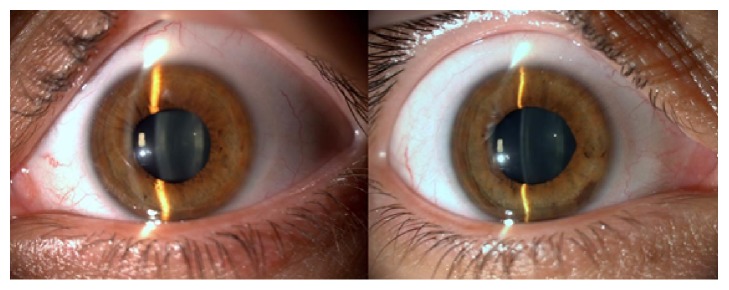
Bilateral iris atrophy correlated with the ablation zone and middilated pupils (due to the probable ischemic damage of the iris sphincter muscle).

**Figure 2:**
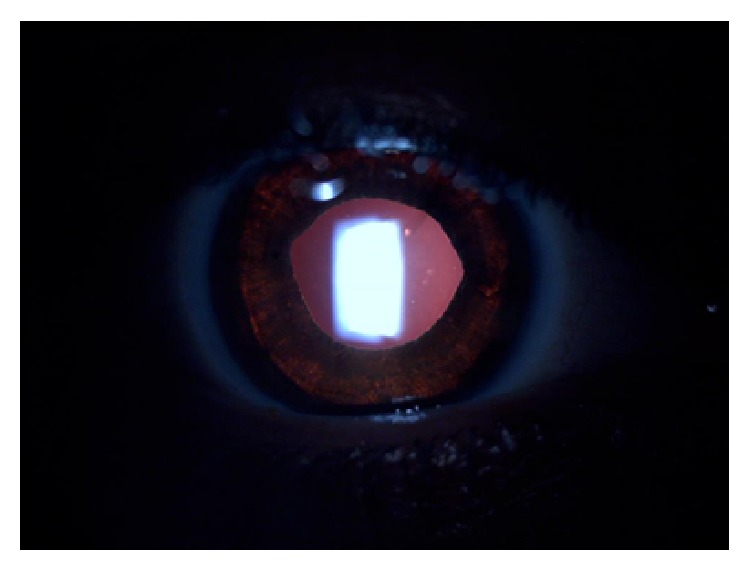
A transillumination defect is remarkable on the biomicroscopic examination.

A 28-year-old female presented with photophobia and glare to our eye service. She stated in her medical history that she had undergone femtosecond assisted LASIK surgery in both eyes 15 months ago and her symptoms started just after this surgery. Preoperative medical records of the patient revealed −4.75 (−1.00 × 175) in the right eye and −4.50 (−0.50 × 180) in the left eye and otherwise a normal ophthalmological examination. On admission, her best-corrected visual acuity was 10/10 in both eyes. She had mydriatic pupils with no direct light reflex. Visual fields were full to confrontation in both eyes. Examination of the anterior segment revealed bilateral iris atrophy projecting within the LASIK ablation zone (Figure 1) and a transillumination defect was remarkable on the slit lamp examination (Figure 2). Funduscopy and intraocular pressures (IOP) were normal in both eyes.

## 3. Discussion

Several flap-related or intraoperative complications of femtosecond assisted LASIK surgery are reported in the literature [1]. However, there is no case presented with such a clinical situation, to the best of our knowledge. Many studies have investigated the effects of LASIK surgery on intraocular pressure and ocular blood flow [2–6]. In a study ocular blood flow changes following LASIK were evaluated using color Doppler imaging and a highly significant decrease in the peak systolic volume and end-diastolic volume of the ophthalmic artery at 1 day and 1 week postoperatively was reported [2]. Yang et al. evaluated the effect of intraocular pressure on blood flow velocity and resistance in the rabbit ophthalmic artery, reporting that the ophthalmic artery in rabbits was capable of maintaining normal blood velocity and resistance when IOP was below 40 mmHg. However, the autoregulatory capacity was greatly limited when IOP was over 40 mmHg [7]. Vetter et al. compared the increase in intraocular pressure (IOP) during corneal flap preparation in porcine eyes when using a femtosecond laser or a mechanical microkeratome and reported that during the worst-case procedure (the eye interface was lowered against the globe until abortion of the docking maneuver when using the IntraLase femtosecond laser or the suction ring was pressed very firmly against the globe when using the Amadeus microkeratome), a maximum IOP of 260 ± 53 mmHg was reached with the IntraLase and 318 ± 59 mmHg was reached with the Amadeus microkeratome [3]. Vetter et al. also reported that the IOP may be elevated to the range of 299.1 to 341.2 mmHg during the worst-case procedure (femtosecond laser interface was pressed against globe until docking maneuver was aborted) with the 60 kHz femtosecond lasers in human donor eyes [8]. In another study, real-time intraocular pressure was compared between laser in situ keratomileusis (LASIK) and epithelial LASIK (epi-LASIK) in porcine eyes during flap creation using a microkeratome or an epikeratome, respectively. In the LASIK group, the mean IOP was 113.65 ± 10.78 during suctioning and 112.35 ± 11.51 mmHg during cutting phases and in the epi-LASIK group, the mean IOP was 92.57 ± 20.86 mmHg during suctioning, 82.09 ± 20 mmHg during cutting phases [4].

All these studies show the significant effects of LASIK surgery on intraocular pressure and ocular hemodynamics. In the literature optic neuropathy cases have been reported following LASIK surgery and it has been considered that this complication might be a result of barotrauma or ischemia related to extremely elevated intraocular pressure during a portion of the LASIK procedure [9–11]. Therefore, we hypothesized that bilateral iris atrophy may have been caused by the abnormally increased IOP that resulted in ischemia in the iris vascular plexus during the suction process in our case.
